# The Expression of Rab8, Ezrin, Radixin and Moesin in the Ciliary Body of Cynomolgus Monkeys

**DOI:** 10.14789/jmj.JMJ21-0042-OA

**Published:** 2022-07-14

**Authors:** KAZUHIKO TANABE, ITARU KIMURA, HARU OKAMOTO, ZAI-LONG CHI, MASAKAZU AKAHORI, NOBUHIRO SHIMOZAWA, NOBUYUKI EBIHARA, AKIRA MURAKAMI, TAKESHI IWATA

**Affiliations:** 1 National Institute of Sensory Organs, National Hospital Organization Tokyo Medical Center, Tokyo, Japan; 1 National Institute of Sensory Organs, National Hospital Organization Tokyo Medical Center, Tokyo, Japan; 2 Department of Ophthalmology, Graduate School of Medicine, Juntendo University, Tokyo, Japan; 2 Department of Ophthalmology, Graduate School of Medicine, Juntendo University, Tokyo, Japan; 3 Department of Ophthalmology, Juntendo University Urayasu Hospital, Chiba, Japan; 3 Department of Ophthalmology, Juntendo University Urayasu Hospital, Chiba, Japan; 4 Tsukuba Primate Research Center, National Institute of Biomedical Innovation, Ibaraki, Japan; 4 Tsukuba Primate Research Center, National Institute of Biomedical Innovation, Ibaraki, Japan

**Keywords:** ciliary body, rab8, ezrin, radixin, moesin

## Abstract

**Purpose:**

The purpose of this study was to determine what proteins are present in the ciliary body (CB). To accomplish this, we conducted a proteomic analysis of the CB of cynomolgus monkeys. We also determined the location of the proteins in CB by immunohistology.

**Methods:**

The eyes of euthanized cynomolgus monkeys were enucleated, and the CB, were isolated from the eyes. Proteins were extracted from the CB and determined by liquid chromatography-mass spectrometry. Separated CB epithelial cells were cultured, and the proteins expressed in the CB were determined by Western blotting. The location of these proteins in the CB was determined by immunohistochemical staining. We also investigated whether adding dexamethasone to the culture medium changed protein expression by the epithelial cells.

**Results:**

Proteomic analysis of the CBs showed that 813 proteins were expressed in the epithelium and stroma. These proteins included the small guanosine triphosphate-binding protein Rab8 and the ezrin/radixin/moesin (ERM) family. Tissue and immunohistological staining confirmed the colocalization of these proteins in non-pigmented CB epithelium. Western blotting of cultured CB epithelial cell lysates showed a tendency that adding dexamethasone changed Rab8 protein expression levels.

**Conclusions:**

Proteomic analysis of CBs identified several proteins involved in the transport and secretion of proteins. These proteins may be involved in the production of aqueous humor and protein secretion by the CB.

## Introduction

Glaucoma is a major cause of blindness worldwide: Approximately 60 million people have glaucoma, and approximately 8 million of them are blind in both eyes^1, 2)^. The disease is characterized by a progressive loss of retinal ganglion cells, resulting in constriction of the visual fields^2, 3)^. Elevated intraocular pressure (IOP) enhances the progression of the disease processes, and a reduction of IOP can slow or block the progression of glaucoma. The IOP can also affect aqueous humor dynamics^4)^. Other components that influence IOP are the trabecular meshwork and Schlemm's canal, which are involved in the outflow of aqueous humor, and the ciliary body (CB), which is involved in aqueous humor production.

Besides aqueous humor formation, the CB is involved in accommodation and anterior chamber-associated immune deviation. The CB, especially the non-pigmented ciliary epithelium, is also involved in the synthesis and secretion of various proteins found in the aqueous humor that are believed to be involved in controlling IOP^4-6)^.

Proteins secreted from the CB can affect the trabecular meshwork cells. The glycoproteins in aqueous humor have been shown to be secreted from the CB epithelium^7)^, and molecules in the aqueous humor, e.g., collagenases, can affect the trabecular meshwork morphology^8, 9)^. However, a comprehensive description of the proteins present in the aqueous humor has not been published.

Recent advances in proteomics technology have made it possible to perform comprehensive investigations of the proteins in body fluids and tissues. In ophthalmology, proteomics studies have analyzed the proteins in the cells of the trabecular meshwork, aqueous humor, retina, retinal pigment epithelium, and retinal drusen^10-17)^.

To date, no study has comprehensively determined the proteins expressed in the CB. In addition, the many steps involved in the synthesis and secretion of aqueous humor have not been determined. Therefore, the purpose of this study was to determine the proteins present in the CB. To accomplish this, we conducted a proteomic analysis of the CB of cynomolgus monkeys and thereby focused on the small guanosine triphosphate (GTP)-binding protein Rab8, which has been shown to be involved in vesicle transport and protein localization in small intestinal epithelial cells. Rab8 is also involved in the morphology of the microvilli in small intestinal epithelial^18)^ and is associated with optineurin^19, 20)^, a glaucoma gene.

## Materials and methods

### Preparation of cynomolgus monkey eyes

All experimental procedures were approved by the Animal Welfare and Animal Care Committee of the Tsukuba Primate Research Center (TRPC) and the Experimental Animal Committee of the National Tokyo Medical Center. The facilities for housing the monkeys are accredited by the Association for Assessment and Accreditation of Laboratory Animal Care International (AAALAC International). Monkeys are routinely examined for physical and ophthalmic conditions by veterinarians and ophthalmologists, and all experiments on monkeys are conducted in accordance with The Association for Research in Vision and Ophthalmology Statement for the Use of Animals in Ophthalmic and Vision Research.

Eyes were obtained in a collaborative study to make effective use of all the monkey tissues for different research programs. Eyes were enucleated immediately after the monkeys were euthanized, and the CBs were isolated from the eyes within 4 hours after death.

### Protein extraction from CBs

The tissues from the CBs of a healthy male monkey were homogenized and sonified in lysis buffer (50 mM Tris-HCL, 2 mM EDTA, 0.5% Triton-X, 2% SDS). After centrifugation for 10 minutes at 10 000 rpm (9,300g), the supernatant was collected. The protein concentration in the supernatant was determined with the RC DC protein assay kit (Bio-Rad Laboratories, Hercules, CA, USA), according to the manufacturer's instructions. Twenty micrograms of the protein sample were combined with an equal volume of 2 × Laemmli buffer and heated for 5 minutes at 100 °C. Then, all samples were stored at -20 °C until use.

### Gel digestion and liquid chromatography-mass spectrometry analyses

Twenty micrograms of protein sample was separated on 12.5% acrylamide SDS-PAGE gel. The gel was stained with Colloidal Coomassie Blue (Invitrogen, Carlsbad, CA, USA) and cut into 15 equal pieces of approximately 1 mm^3^. The pieces were washed twice with 50mM ammonium bicarbonate/50% acetonitrile, and after destaining, the gel pieces were rinsed with distilled water and incubated with acetonitrile for 20 minutes. The supernatant was discarded, and the gel pieces were completely dried before incubation with 10mM DTT in 100mM ammonium bicarbonate for 45 minutes at 56 °C. The supernatant was discarded, and the pieces were incubated in the dark with 55mM iodoacetamide in 100mM ammonium bicarbonate for 30 minutes at room temperature. Then, the supernatant was discarded, and the gels were washed three times. Finally, the gel pieces were completely dried before tryptic digestion in sequencing grade trypsin solution (12.5 μg/μL; Thermo Fisher Scientific Inc, Rockford, IL, USA) in 50mM ammonium bicarbonate. The digestion was performed at 37 °C overnight, and then the extraction step was performed once with 25mM ammonium bicarbonate, twice with 5% formic acid, and then once with distilled water. The extracted peptides were pooled and dried. After re-suspending in 40 μL of aqueous 0.01% trifluoroacetic acid/2% acetonitrile, the samples were analyzed by liquid chromatography-mass spectrometry (LCQ Deca XP plus, Thermo Fisher Scientific Inc).

### Proteomic analysis of monkey CB

Database searches were performed with the assistance of Bio Works 3.3.1, a protein search program. The UniProt-SwissProt database was initially used by querying the entire theoretical peptide masses provided in the public domain by the Swiss Institute of Bioinformatics. The number of registrations in the UniProt-SwissProt database is about 17 000 for humans and about 14 000 for mice. The data analyses of the monkey CB were aimed at cynomolgus monkeys, humans, orangutans, chimpanzees, rhesus macaques, and lowland gorillas.

### Immunohistochemistry of the CB

Enucleated eyes from normal cynomolgus monkeys were fixed in 10% neutralized and buffered formaldehyde solution at 4 °C overnight and then dehydrated. The eyes were embedded in paraffin and serially sectioned at 4 μm thickness. After deparaffinization and rehydration, the specimens were prepared for antigen retrieval by warming in hot water in Target Retrieval Solution (Dako, Glostrup, Denmark) for 30 minutes at 100 °C. The sections were then blocked with phosphate-buffered saline (PBS) containing 10% BSA for one hour and then incubated overnight with primary antibodies (Abs) that were the same as those used for the Western blotting. The slides were washed in PBS and, for nuclear staining, were incubated with Alexa 488 or Alexa 568 (1:500 dilution; Invitrogen) and 4',6'- diamidino-2-phenylindole (DAPI) for one hour at room temperature. The stained tissues were examined with a confocal fluorescence laser microscope (Radiance 2000, Bio-Rad Laboratories). Control slides were prepared by a similar process, but the primary Abs were omitted.

### Primary culture of ciliary epithelial cells from cynomolgus monkey

The ciliary epithelial cells isolated from cynomolgus monkey eyes were suspended in DMEM (GIBCO, Carlsbad, CA, USA) containing penicillin-streptomycin (100 U/mL final concentration; Invitrogen) and 10% FBS. Cells were grown to confluence at 37 °C in 5% CO_2_. Then, cells were cultured with 100 nM of the glucocorticoid dexamethasone (DEX), 500 nM of timolol malate, or 1 μM of acetazolamide (Sigma-Aldrich, St Louis, MO, USA) for five days.

### Protein expression of lysate from blotting of CB

Cultured monkey ciliary epithelial cells were lysed in TNE buffer containing 50mM Tris, 137mM NaCL, 1mM EDTA, 1% TritonX-100, and protease inhibitors (Complete EDTA-free; Roche, Basel, Switzerland). After homogenization and centrifugation for 15 minutes at 14 000 rpm, the supernatant was collected. The protein concentration was determined with the RC-DC protein assay kit (Bio-Rad) according to the manufacturer's instructions. Ten micrograms of proteins from the monkey ciliary epithelium cells lysates were diluted in an equal volume of 2×Laemmli buffer and heated for 5 minutes at 100 °C. Samples were separated by 7.5% SDS-PAGE and transferred electrophoretically to polyvinylidene difluoride membrane. Membranes were blocked in PBS containing 0.05% tween20 (PBS-T) and 5% non-fat dry milk for one hour and probed overnight at 4 °C with one of the following primary Abs: rabbit anti-Rab8 Ab (Sigma-Aldrich), goat anti-Ezrin Ab (Sigma-Aldrich), goat anti-Radixin Ab (Sigma-Aldrich), goat anti-Moesin Ab (Sigma), and mouse anti-Actin Ab (Millipore, Billerica, MA, USA). The specific signals were detected with horseradish peroxidase (HRP)- conjugated donkey Ab to goat IgG or rabbit IgG as secondary Abs. The signals were made visible by chemiluminescence reactions and examined with a ChemiDoc XRS plus (Bio-Rad).

## Results

### Proteomic analysis of monkey CB

**Figure 1:**
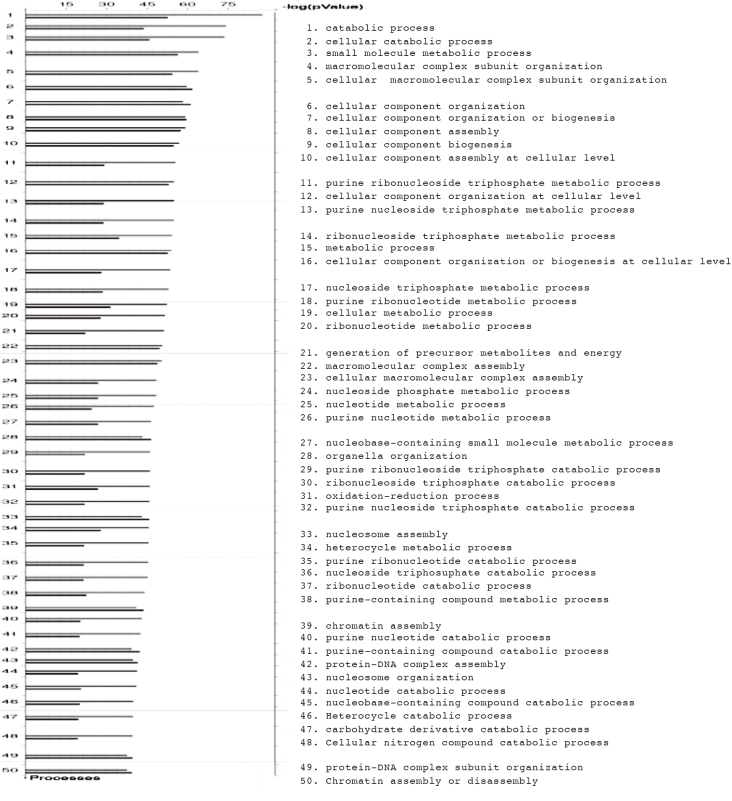
Classification according to the Gene Ontology database The pathway tool software (MetaCore,Gene GO,Infocom) was used to classify all of the identified proteins by the biological process registered in the Gene Ontology database. Upper bar, proteins identified mainly in the ciliary epithelium; lower bar, proteins identified mainly in the ciliary stroma

The proteomic analysis of proteins extracted from the stroma and epithelium of the monkey CB identified a total of 813 proteins. Detailed information on these proteins is shown as supplemental data. These proteins were classified by a biological process registered in the Gene Ontology database that uses pathway tool software (MetaCore, Gene GO, Infocom; Figure 1). The proteins identified in the CB are involved in protein transport, localization, and secretion (Table 1).

**Table 1:** Principal proteins of cynomolgus monkey ciliary body

No. Protein name	Database	MW	Sequence	No.
	accession No.	(Da)	coverage(%)	of peptide
1 10 kDa heat shock protein, mitochondrial	P61604	10924.9	25.50	3
2 14 kDa phosphohistidine phosphatase	Q9NRX4	13823.7	16.80	1
3 14-3-3 protein beta/alpha	P31946	28064.8	10.20	5
4 40S ribosomal protein S10	P46783	18885.9	5.50	2
5 60 kDa heat shock protein, mitochondrial	P10809	61016.5	17.60	10
6 60S acidic ribosomal protein P0	P05388	34251.8	3.50	2
7 78 kDa glucose-regulated protein	P11021	72288.5	13.80	7
8 Acetylcholinesterase	P22303	67753.4	2.10	1
9 Aconitate hydratase, mitochondrial	Q99798	85372.0	19.90	31
10 Actin, alpha cardiac muscle 1	P68032	41991.9	46.40	181
11 Acyl-protein thioesterase 1	O75608	24653.5	6.10	1
12 ADAM 21	Q9UKJ8	80766.1	1.10	1
13 Adenomatous polyposis coli protein	P25054	311453.1	0.50	1
14 Adenosylhomocysteinase	P23526	47685.3	3.50	1
15 ADP/ATP translocase 1	P12235	33043.2	17.80	28
16 Aldose 1-epimerase	Q96C23	37742.1	4.10	1
17 Alpha-2-macroglobulin	P01023	163174.3	7.60	16
18 Alpha-actinin-1	P12814	102992.7	20.50	30
19 Alpha-adducin	P35611	80904.8	2.40	1
20 Alpha-crystallin A chain	P02489	19896.9	31.20	5
21 Alpha-enolase	P06733	47139.4	24.40	34
22 Alpha-internexin	Q16352	55357.5	2.40	8
23 Annexin A1	P04083	38690.0	20.20	6
24 Apolipoprotein O	Q9BUR5	22270.5	23.20	3
25 Arachidonate 5-lipoxygenase	P09917	77933.4	2.10	2
26 Aspartate aminotransferase, cytoplasmic	P17174	46218.6	11.10	4
27 ATP synthase subunit alpha, mitochondrial	P25705	59713.7	21.00	32
28 Beta-actin-like protein 2	Q562R1	41976.0	12.00	92
29 Beta-crystallin A3	P05813	25133.8	7.00	1
30 Beta-enolase	P13929	46957.4	11.10	11
31 Calcium-binding mitochondrial carrier protein Aralar1	O75746	74709.0	4.90	4
32 Calnexin	P27824	67526.0	9.10	3
33 Calponin-1	P51911	33149.6	10.40	4
34 Calreticulin	P27797	48111.9	4.30	2
35 Carbonic anhydrase 2	P00918	29227.9	3.50	1
36 Caskin-2	Q8WXE0	126633.8	0.00	1
37 Catenin alpha-1	P35221	100008.6	2.00	1
38 Cationic amino acid transporter 3	Q8WY07	67125.6	1.90	2
39 CD59 glycoprotein	P13987	14167.8	8.60	4
40 CD9 antigen	P21926	25399.0	5.30	2
41 Centromere protein C 1	Q03188	106860.4	1.30	1
42 Citrate synthase, mitochondrial	O75390	51679.6	7.70	4
43 Clathrin heavy chain 1	Q00610	191491.7	6.30	12
44 Coatomer subunit alpha	P53621	138243.8	1.00	1
45 Cofilin-1	P23528	18490.7	28.30	9
46 Complement C3	P01024	187029.3	1.00	1
47 Contactin-2	Q02246	113322.6	1.40	1
48 Creatine kinase B-type	P12277	42617.4	32.50	21
49 Cystatin-B	P04080	11132.6	12.20	1
50 Cytochrome c	P99999	11741.1	33.30	13
51 Cytoplasmic aconitate hydratase	P21399	98336.7	1.60	1
52 Cytoskeleton-associated protein 5	Q14008	225364.6	0.80	1
53 Dachshund homolog 1	Q9UI36	78513.4	2.00	1
54 D-dopachrome decarboxylase	P30046	12703.7	9.30	2
55 Decorin	P07585	39721.9	4.20	1
56 Density-regulated protein	O43583	22078.1	6.60	1
57 Desmin	P17661	53503.2	39.40	65
58 Destrin	P60981	18493.5	28.50	10
59 Diacylglycerol kinase epsilon	P52429	63885.0	0.00	1
60 Dihydropteridine reductase	P09417	25773.0	12.30	2
61 Dihydropyrimidinase-related protein 1	Q14194	62144.8	2.80	1
62 DmX-like protein 2	Q8TDJ6	339541.7	0.50	1
63 DNA mismatch repair protein Msh2	P43246	104676.8	0.00	1
64 DNA polymerase kappa	Q9UBT6	98745.8	1.70	1
65 Dynamin-2	P50570	98003.3	1.60	2
66 Dynein heavy chain 5, axonemal	Q8TE73	528683.8	0.30	1
67 Dysferlin	O75923	237142.3	0.00	1
68 Dystrophin	P11532	426426.0	0.40	2
69 Electron transfer flavoprotein subunit alpha, mitochondrial	P13804	35057.6	8.70	2
70 Elongation factor 1-alpha 1	P68104	50109.2	7.10	6
71 Endoplasmin	P14625	92411.2	2.40	2
72 Endothelin-converting enzyme-like 1	O95672	87735.7	3.40	2
73 Eyes absent homolog 3	Q99504	62519.0	3.10	1
74 Ezrin	P15311	69369.8	3.20	2
75 Ferritin heavy chain	P02794	21212.3	13.10	6
76 Fibrillin-1	P35555	312097.0	0.50	1
77 Filamin-A	P21333	280561.4	2.80	11
78 Fructose-bisphosphate aldolase A	P04075	39395.3	9.30	5
79 Fumarate hydratase, mitochondrial	P07954	54602.2	7.30	2
80 Fumarylacetoacetate hydrolase domain-containing protein 2A	Q96GK7	34574.1	16.60	5
81 Galectin-1	P09382	14706.2	32.60	5
82 Gamma-enolase	P09104	47239.1	7.60	5
83 Gelsolin	P06396	85644.3	14.10	16
84 Glucose-6-phosphate isomerase	P06744	63107.3	11.50	5
85 Glutamate receptor 2	P42262	98758.3	1.80	3
86 Glutathione S-transferase kappa 1	Q9Y2Q3	25480.3	10.60	3
87 Glyceraldehyde-3-phosphate dehydrogenase	P04406	36030.4	40.00	63
88 Haptoglobin	P00738	45176.6	13.30	7
89 Heat shock 70 kDa protein 1	P08107	70009.2	9.00	9
90 Hemoglobin subunit alpha	P69905	15247.9	23.90	10
91 Hexokinase-1	P19367	102420.2	3.20	3
92 Histone H4	P62805	11360.4	53.40	22
93 Ig alpha-1 chain C region	P01876	37630.7	15.60	4
94 Inactive phospholipase C-like protein 2	Q9UPR0	125785.3	1.00	6
95 Interphotoreceptor retinoid-binding protein	P10745	135277.8	1.10	1
96 Kallikrein-12	Q9UKR0	26716.2	6.50	1
97 Kappa-actin	Q9BYX7	41988.9	4.30	64
98 Kelch-like protein 8	Q9P2G9	68757.9	1.60	1
99 Kinesin-like protein KIF23	Q02241	109989.7	0.70	1
100 Lactoylglutathione lyase	Q04760	20764.3	4.30	1
101 Lamin-A/C	P02545	74094.8	21.80	30
102 Laminin subunit alpha-4	Q16363	202399.2	0.90	2
103 L-lactate dehydrogenase A chain	P00338	36665.4	3.60	2
104 Lutheran blood group glycoprotein	P50895	67362.7	4.80	8
105 Macrophage migration inhibitory factor	P14174	12468.2	7.80	2
106 Malate dehydrogenase, cytoplasmic	P40925	36403.0	23.10	18
107 Microsomal glutathione S-transferase 3	O14880	16505.6	17.80	3
108 Moesin	P26038	67777.9	5.50	2
109 Mu-crystallin homolog	Q14894	33754.4	7.30	2
110 Myelin basic protein	P02686	33097.3	11.20	7
111 Myosin-1	P12882	222975.5	0.50	3
112 Myotrophin	P58546	12886.6	30.50	2
113 Nesprin-1	Q8NF91	1010433.0	0.10	1
114 Nestin	P48681	177331.0	1.10	1
115 Neuroblastoma-amplified gene protein	A2RRP1	268412.5	0.00	1
116 Neurofilament heavy polypeptide	P12036	112412.0	0.90	8
117 Neurolysin, mitochondrial	Q9BYT8	80599.8	0.00	2
118 Neutral alpha-glucosidase AB	Q14697	106806.8	2.40	1
119 Nucleolar protein 14	P78316	97607.5	1.50	1
120 Nucleoside diphosphate kinase 3	Q13232	19002.9	10.10	2
121 Ovochymase-1	Q7RTY7	124985.6	1.10	1
122 P2X purinoceptor 1	P51575	44951.2	3.80	1
123 Palmitoyl-protein thioesterase 1	P50897	34171.3	7.80	1
124 Peptidyl-prolyl cis-trans isomerase A	P62937	18000.9	64.20	29
125 Periaxin	Q9BXM0	154906.0	4.90	2
126 Peripherin	P41219	53618.5	6.60	16
127 Peroxiredoxin-1	Q06830	22096.3	24.60	6
128 Phosphate carrier protein, mitochondrial	Q00325	40068.8	9.90	19
129 Phosphatidylethanolamine-binding protein 1	P30086	21043.7	27.30	11
130 Phosphoglucomutase-1	P36871	61410.6	5.70	3
131 Phosphoglycerate mutase 1	P18669	28785.9	31.50	12
132 Plasma membrane calcium-transporting ATPase 3	Q16720	134112.2	1.50	1
133 Plasminogen	P00747	90510.2	2.10	1
134 Plectin-1	Q15149	531407.9	0.30	1
135 Potassium channel subfamily K member 3	O14649	43490.0	0.00	1
136 Pre-B-cell leukemia transcription factor-interacting protein 1	Q96AQ6	80594.2	4.20	3
137 Pregnancy zone protein	P20742	163728.1	1.50	4
138 Prenylcysteine oxidase 1	Q9UHG3	56603.8	3.20	1
139 Proactivator polypeptide	P07602	58073.9	2.10	1
140 Probable phosphoglycerate mutase 4	Q8N0Y7	28758.8	11.80	7
141 Profilin-2	P35080	15036.3	10.00	2
142 Prohibitin-2	Q99623	33275.9	14.00	4
143 Prolargin	P51888	43782.2	2.90	1
144 Prolyl endopeptidase	P48147	80712.1	1.80	1
145 Prostaglandin E synthase 3	Q15185	18685.4	6.30	1
146 Proteasome subunit beta type-3	P49720	22933.5	7.80	1
147 Protein AF-10	P55197	108958.5	1.10	1
148 Protein disulfide-isomerase	P07237	57080.8	3.10	3
149 Protein DJ-1	Q99497	19878.5	7.90	3
150 Protocadherin-15	Q96QU1	215932.5	0.80	1
151 Putative annexin A2-like protein	A6NMY6	38634.8	11.20	14
152 Putative elongation factor 1-alpha-like 3	Q5VTE0	50153.2	4.80	6
153 Putative GTP-binding protein RAY-like	Q9BW83	20467.3	4.30	1
154 Putative heat shock 70 kDa protein 7	P48741	40219.6	3.50	2
155 Putative histone H2B type 2-C	Q6DN03	21458.2	5.20	7
156 Putative nucleoside diphosphate kinase	O60361	15519.0	33.60	6
157 Putative RNA methyltransferase NOL1	P46087	89247.2	1.40	1
158 Putative tubulin beta-4q chain	Q99867	48403.5	5.80	8
159 Pyruvate kinase isozymes M1/M2	P14618	57900.2	35.20	32
160 Radixin	P35241	68521.5	5.80	3
161 Ras suppressor protein 1	Q15404	31520.7	6.10	1
162 Ras-related C3 botulinum toxin substrate 1	P63000	21436.3	5.20	1
163 Ras-related protein Rab-10	P61026	22526.6	6.00	1
164 Ras-related protein Rab-11A	P62491	24378.4	14.40	4
165 Ras-related protein Rab-1A	P62820	22663.4	16.10	2
166 Ras-related protein Rab-2A	P61019	23530.8	6.60	1
167 Ras-related protein Rab-3A	P20336	24968.1	7.30	1
168 Ras-related protein Rab-5C	P51148	23467.8	6.50	1
169 Ras-related protein Rab-7a	P51149	23474.9	6.80	1
170 Ras-related protein Rab-8A	P61006	23653.2	6.80	1
171 Ras-related protein Rap-1A	P62834	20973.7	13.60	3
172 Ras-related protein Rap-1b	P61224	20811.6	13.60	3
173 Retinal dehydrogenase 1	P00352	54827.0	3.00	1
174 Rho GDP-dissociation inhibitor 1	P52565	23192.7	7.40	6
175 RNA-binding protein 44	Q6ZP01	117910.9	1.30	2
176 Secernin-1	Q12765	46352.6	3.10	1
177 Selenium-binding protein 1	Q13228	52357.7	16.10	4
178 Septin-2	Q15019	41461.3	8.00	2
179 Serotransferrin	P02787	76999.7	17.80	12
180 Serum albumin	P02768	69321.6	22.50	74
181 Sideroflexin-3	Q9BWM7	35480.5	4.70	1
182 Signal peptide peptidase-like 2C	Q8IUH8	74404.0	2.20	2
183 Sodium/potassium-transporting ATPase subunit alpha-1	P05023	112824.1	8.70	15
184 Solute carrier family 2, facilitated glucose transporter member 1	P11166	54048.7	2.00	3
185 Sorcin	P30626	21662.4	5.60	1
186 Spectrin alpha chain, brain	Q13813	284362.5	7.00	18
187 Stomatin-like protein 2	Q9UJZ1	38510.2	4.20	2
188 Stress-70 protein, mitochondrial	P38646	73634.8	16.60	10
189 Superoxide dismutase [Mn], mitochondrial	P04179	24706.6	16.20	3
190 Talin-1	Q9Y490	269596.3	1.60	5
191 Tektin-4	Q8WW24	50617.3	0.00	1
192 Thioredoxin-dependent peroxide reductase, mitochondrial	P30048	27675.2	12.10	4
193 Titin	Q8WZ42	3812906.0	0.00	1
194 Transgelin	Q01995	22596.4	16.40	6
195 Transketolase	P29401	67834.9	6.40	8
196 Translationally-controlled tumor protein	P13693	19582.6	15.10	2
197 Trichoplein keratin filament-binding protein	Q9BT92	61034.3	2.40	2
198 Trifunctional enzyme subunit alpha, mitochondrial	P40939	82947.0	8.50	8
199 Triosephosphate isomerase	P60174	26652.7	57.00	26
200 Tropomyosin alpha-1 chain	P09493	32688.7	27.50	22
201 Tubulin alpha-1A chain	Q71U36	50103.7	16.20	25
202 Tyrosine aminotransferase	P17735	50366.5	3.10	1
203 Ubiquitin	P62988	8559.6	32.90	8
204 Vacuolar proton pump subunit E 1	P36543	26128.8	6.20	1
205 Vesicle-associated membrane protein 2	P63027	12640.7	20.70	6
206 Vimentin	P08670	53619.2	52.10	134
207 Vinculin	P18206	123721.9	3.50	4
208 Voltage-dependent anion-selective channel protein 1	P21796	30753.6	23.00	6
209 Wolframin	O76024	100241.2	1.50	1
210 Zinc finger protein 577	Q9BSK1	54122.0	3.30	1

### Immunohistochemical analyses

**Figure 2:**
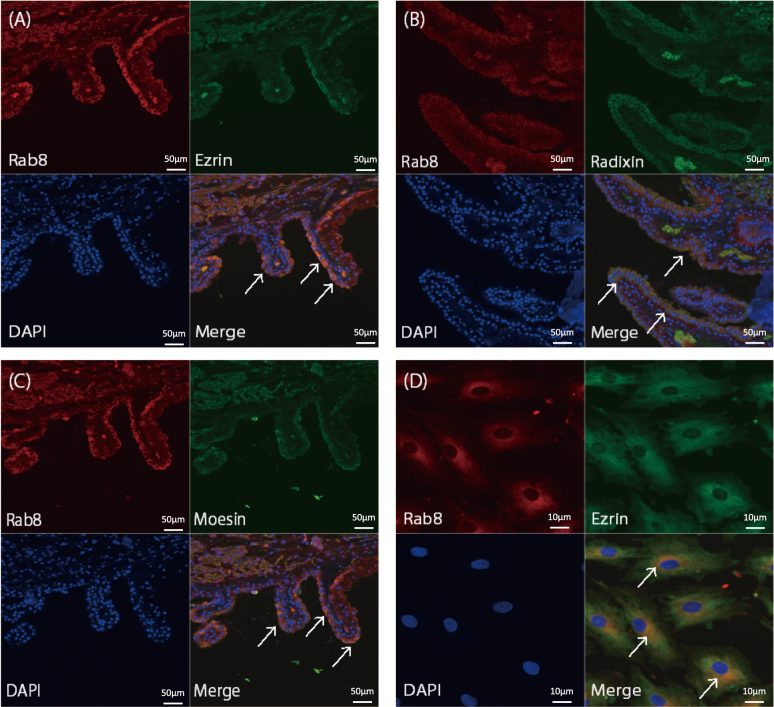
Immunohistochemistry of normal cynomolgus monkey ciliary body, and immunofluorescence staining of cultured monkey ciliary body cells Paraffin sections of cynomolgus monkey ciliary body (CB) were labeled with antibodies specific to Rab8 (A-C), ezrin (A), radixin (B), and moesin (C). Monkey CB cells were stained with Rab8 and ezrin (D). Co-expression of Rab8 and the ezrin/radixin/moesin (ERM) family can be seen at the ciliary non-pigmented epithelium cells (arrow).

The results of proteomic analysis of monkey CB included the Rab8 protein (No.170), which is a small GTP-binding protein that is related to the glaucoma gene optineurin, and is known to be involved in protein localization and transportation in the small intestine and can affect the morphology of microvilli^18)^. The ezrin (No.70)/radixin (No.160)/moesin (No.108), ERM family, which are core proteins in the microvilli that also act as linker proteins between actin filaments and the cell membranes. Rab8 and moesin have also been reported to interact in the photoreceptor cells^21)^. It is reasonable to assume that Rab8 and ERM family interact also in CB, and are involved in protein secretion. Double immunostaining with anti-Rab8 Ab and anti-ERM family Abs showed expression of Rab8, ezrin, radixin, and moesin in the CB epithelium and especially in the non-pigmented epithelium. A co-expression of Rab8 and the ERM family proteins was also detected in the ciliary epithelium (Figure 2A, 2B, 2C) and primary cultured ciliary epithelial cells (Figure 2D).

### Drug effects on ciliary epithelium cells in vitro

**Figure 3:**
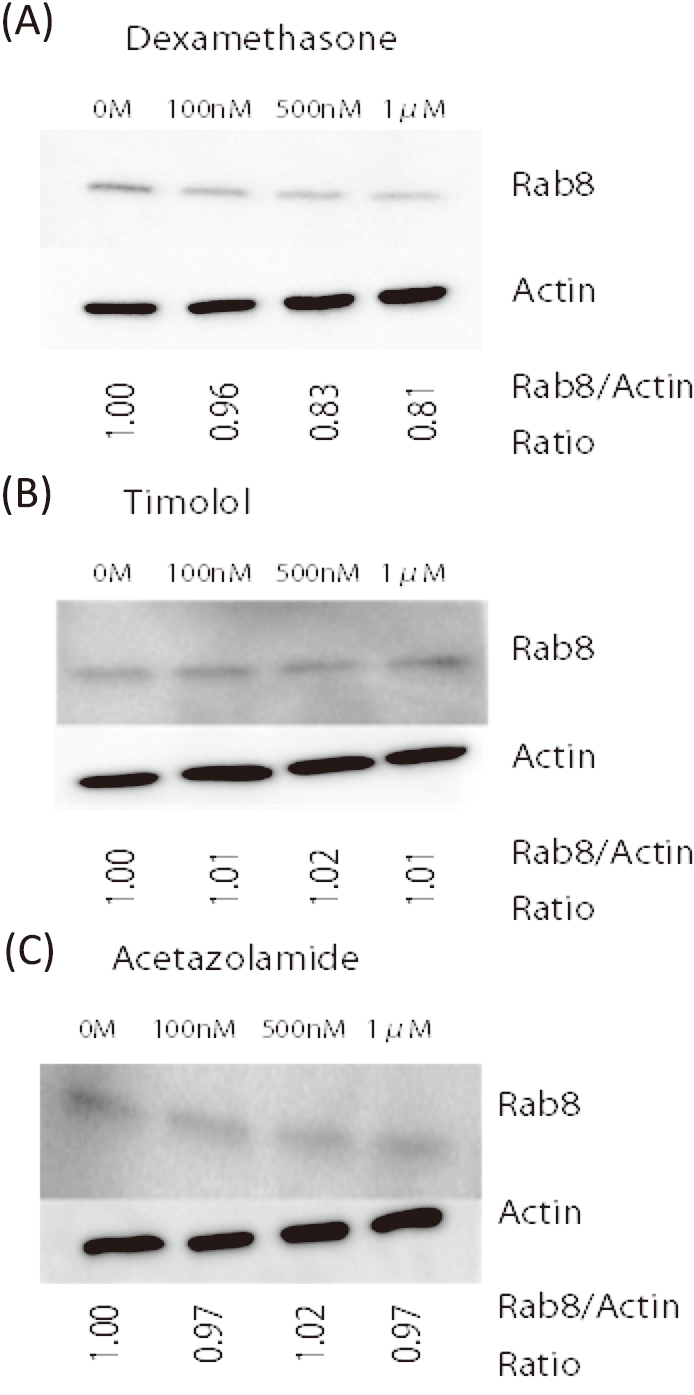
Effect of dexamethasone on the expression of Rab8 in monkey ciliary epithelial cells A: Western blot analysis with an antibody against Rab8 showed a tendency of decrease in the expression of Rab8 in monkey ciliary epithelial cells after exposure to dexamethasone (Dex) for 5 days. Western blot analysis with an antibody against actin was the control. The relative amount of Rab8/actin was quantified. B: Western blot analysis of ciliary epithelial cells after exposure to timolol maleate for five days. No change was seen in the expression of Rab8 proteins. C: Western blot analysis of ciliary epithelial cells after exposure to acetazolamide for five days. No change was seen in the expression of Rab8 proteins.

The secondary glaucoma caused by glucocorticoids is both clinically and morphologically similar to primary open angle glaucoma. When trabecular meshwork cells are exposed to DEX, the extracellular matrix increases and the trabecular meshwork thickens, and resulting in IOP elevation. It is known that glucocorticoids affect IOP. However, the relationship between CB function and glucocorticoids remains unclear. We investigated whether Rab8 interacts the mechanism of IOP elevation by glucocorticoids stimulation. Light microscopy did not show any morphological changes in the morphology and protein expression of cultured cynomolgus monkey non-pigmented ciliary epithelial cells with different concentrations of DEX(data not shown). Western blotting of epithelial cell lysates showed a tendency for the addition of DEX to decrease the expression of Rab8 protein and this effect seemed dose-dependent, though this is not significant(Figure 3A). The experiments repeated twice and the results were similar. In addition, when cells were cultured in the same way and timolol maleate or acetazolamide was added, no changes were observed in the expression of Rab8(- Figure 3B, 3C).

## Discussion

Proteomic analyses of the CB of cynomolgus monkeys detected many proteins, some of which are involved in protein transport and secretion. In the CB, the difference of protein characteristics was found between ciliary epithelium and stroma. Structure proteins were major component in stroma, and catalytic and metabolic proteins were more expressed in epithelium than stroma. Diffusion, ultrafiltration, and active secretion are physiological processes that participate in the production of aqueous humor, especially active secretion^4)^. Blood flows through the CB, and aqueous humor is produced from the blood plasma by the non-pigmented ciliary epithelium. The proteins in the aqueous humor differ both quantitatively and qualitatively from those in the plasma^4, 22, 23)^, indicating that the CB must be involved in the secretion of proteins into the aqueous humor. In comparison of CB with aqueous humor, Chowdhury et al reported that 355 proteins were identified from human aqueous humor by narrow liquid chromatography electrospray ionization tandem mass spectrometry, and most of the proteins had catalytic, enzymatic, and structural properties^23)^. Catalytic and metabolic proteins are almost 30% each of top 50 distribution by function by Gene Ontology in CB, and approximately 20% each in aqueous humor. Structure proteins take up around 35% in CB, whereas 9.9% in aqueous humor^23)^, though simple comparison is not possible according to different way of classification.

Myocilin, a glaucoma gene, is found in the aqueous humor, and the trabecular meshwork cells are known to secrete exosomes into the aqueous humor^24)^. The CB appears to secrete proteins into the aqueous humor in the same way.

Rab8, one of the proteins identified by our proteomic analysis, is a small GTP-binding protein that is related to optineurin, a glaucoma gene^18-20)^. It is known to be involved in protein localization and transportation in the small intestine and can affect the morphology of microvilli^18)^. Although no reports have described the role that Rab8 plays in the CB, it is quite reasonable to assume that it is involved in protein secretion.

Our proteomic analysis also identified the ERM family proteins, which are core proteins of the microvilli and are found directly beneath the cell membranes. They act as linker proteins and connect actin filaments to the cell membranes^25, 26)^. Rab8 and moesin have been reported to interact to regulate the transport of rhodopsin in photoreceptor cells^21)^. Both proteins appear to be related to protein transport and to be active in the CB.

We double immunostained for Rab8, ezrin, radixin, and moesin in cynomolgus monkey CB tissues and also in cultured non-pigmented ciliary epithelial cells. Confocal microscopy showed a co-expression of Rab8 and the ERM family in the non-pigmented epithelium of the CB, suggesting that these proteins are involved in the function of the non-pigmented epithelium of the CB.

The secondary glaucoma caused by glucocorticoids is both clinically and morphologically similar to primary open angle glaucoma. Thus, glucocorticoids have been used in both *in vivo* and *in vitro* glaucoma research^27-31)^. When trabecular meshwork cells are exposed to DEX, the extracellular matrix increases and the trabecular meshwork thickens, resulting in an elevation of IOP^31)^. It is also widely known that glucocorticoids affect IOP. However, the relationship between CB function and glucocorticoids remains unclear.

We cultured non-pigmented ciliary epithelial cells and found a tendency for adding DEX to reduce the level of Rab8 expression in these cells. Although drugs such as beta-blockers and acetazolamide can alter the production of aqueous humor, we did not observe any changes in the level of Rab8 expression when timolol maleate or acetazolamide was added to cultured CB epithelial cells. We suggest that these proteins influence the function and morphology of the trabecular meshwork. They may also affect the aqueous humor outflow route and thus the IOP. Determining these functions would be relevant for understanding the mechanism of IOP control and glaucoma pathogenesis.

Although Rab8 might be involved in functions in the CB besides the secretion of aqueous humor, the finding that Rab8 expression in the CB was affected by DEX stimulation indicates that Rab8 may have some effect on IOP dynamics. To obtain more information on this relationship, analyses of the CB epithelial cell secretome and secreted exosomes and experiments involving co-culture models with trabecular meshwork cells are required.

In conclusion, Rab8 probably plays some role in the secretion of aqueous humor in the CB of cynomolgus monkeys and co-exists with ERM family molecules. Its expression is dependent on stimulation by glucocorticoids. Further studies will be needed to investigate the exact role of Rab8 in the CB.

## Funding

No funding was received.

## Author contributions

KT and IK collected and analyzed the experimental date, drafted the manuscript. HO, AC, MA and NS supported the experiment, NE, AM and TI conceived and participated in the study design and critically reviewed the manuscript.

## Conflicts of interest statement

The authors declare that there are no conflicts of interest.
